# Patient and Care Team Perspectives of Barriers to and Facilitators for the Implementation of a Digital Health Program for Depression in Primary Care: Qualitative Study

**DOI:** 10.2196/72003

**Published:** 2026-01-29

**Authors:** Andrea Nederveld, Elise A Robertson, Angela M Lanigan, Elisabeth F Callen, Tarin L Clay, Ben Fehnert, Lambros Chrones, Michael L Martin, Margaret McCue, Christina M Hester, Melissa K Filippi

**Affiliations:** 1 University of Colorado Anschutz Medical Campus, Aurora, CO, United States; 2 National Research Network, American Academy of Family Physicians, Leawood, KS, United States; 3 DARTNet Institute, 12635 E Montview Blvd Ste 129, Aurora, CO, 20045, United States, 1 8004340278 ext 78; 4 Ctrl Group/Fora Health, London, United Kingdom; 5 Takeda Pharmaceuticals U.S.A., Inc., Lexington, MA, United States; 6 Robert Graham Center, American Academy of Family Physicians, Washington, DC, United States; 7 Department of Family Medicine, Georgetown University, Washington, DC, United States

**Keywords:** depression treatment, digital health, family physicians, goal setting, depression, major depressive disorder, MDD, mobile health, primary care, qualitative, shared decision-making

## Abstract

**Background:**

Depression is pervasive, and rates are rising in the United States. Most people with depression receive care from primary care clinicians, but gaps in the quality of care exist. Team-based approaches to depression care have been shown to aid in treatment and management; yet, challenges exist in implementation. Digital health apps have been shown to be effective in improving depression symptoms and enhancing patient engagement in some populations. Many, however, do not share data with clinical care teams.

**Objective:**

This study aimed to understand the barriers to and facilitators for implementation of a digital health program that supports coordinated use by clinical care teams and patients, via a mobile app and care team–facing web interface, for depression in primary care.

**Methods:**

This study was part of a larger intervention study that included 4 primary care practices: 2 intervention and 2 control sites. The intervention sites used a patient-facing mobile app and a care team–facing web interface, and the control sites continued usual care. The study team conducted interviews from May to October 2021. Patient and care team participants were recruited toward the end of their study involvement. Separate semistructured interview guides were developed for patient and care team participants. Interviews were recorded and transcribed. Data were coded using Atlas.ti.9, and data analysis was completed using a grounded theory approach.

**Results:**

Interviews with patient (n=8) and care team (n=8) participants revealed 3 main topics for program implementation: app/interface usability, tracking, and program recommendations. For app/interface usability, overall, navigation for both patient and care team participants was simple and straightforward. Although app content was relevant, patient participants desired additional educational resources and information to aid in their depression treatment and management. In terms of tracking, care team participants indicated that data obtained via the interface enabled them to monitor patients in between visits; and in some circumstances, these data facilitated conversations with patients about treatment plans. Tracking medication adherence differed among patient participants due to established routines of taking medications consistently, lack of motivation to track, or lack of interest in tracking. Care team participants reported the ability to respond more quickly to side effects. Patients commented on tracking difficulties: confusing response options, insufficient goal attainment response options, not being able to provide details or write notes, and no ability to change goals. Some patient and care team participants perceived that tracking encouraged communication with one another; others perceived tracking as having no impact on shared decision-making.

**Conclusions:**

Results suggest implementation of a digital health program for depression treatment and management in primary care practices could impact patient medication adherence, produce faster turnaround time for medication optimization, encourage goal setting, and foster communication between patients and care team members. Program enhancements could optimize patient and care team member engagement.

## Introduction

Primary care clinical teams play a critical role in depression diagnosis, treatment, and management. Rates of depression in the United States have risen since the beginning of the COVID-19 pandemic [1]. Up to 70% of patients being treated for behavioral and mental health conditions, including depression, receive care within primary care [2], and approximately 13% of primary care visits involved a patient with a depression diagnosis between 2010 and 2018 [3]. Clinician comfort with treating depression varies [4], and gaps in quality of care exist; notably in areas such as screening, diagnosis, patient engagement and education, clinician training, and clinician follow-up [5-10]. There is evidence that team-based care and care management between clinic visits, as well as patient self-management [11], can improve depression outcomes [12,13]. However, these strategies can be time and resource intensive for primary care practices, requiring regular check-ins with patients and providing education on self-management. Encouraging patients to use digital health apps is one strategy to assist patients in better self-management.

Health care providers can play an integral role in patient initiation of use of a digital or mobile app [14]. Digital health apps are acceptable to patients, easy to use, and potentially effective in supporting behavior change and self-management, particularly in improving depression and anxiety symptoms in various populations [15-19]. However, there is a lack of standardization in evaluating health apps [20,21], and many behavioral health apps are not able to share data with clinical care teams [20].

This pilot study examined a digital health program that included a patient-facing mobile app and care team web interface that support depression treatment and symptom management in relation to shared decision-making and goal setting in primary care practices. Here, we describe patient and care team participant perspectives regarding barriers to and facilitators for using the digital health program in their practices.

## Methods

### Study Design, Setting, and Recruitment

This study was part of a larger intervention study (currently under review) that included 4 primary care practices, located in Michigan (n=1), Florida (n=1), and New York (n=2), all with some level of behavioral health integration. The intervention arm (n=2 practices, 1 residency and 1 private practice) piloted the use of the Primary Care Path, a program with a patient-facing mobile app and accompanying care team–facing web interface, which was developed collaboratively by Takeda and American Academy of Family Physicians National Research Network (AAFP NRN) and powered by Fora Health (Ctrl Group Limited UK). The control arm (n=2 practices; 1 Community Health Center and 1 private practice) continued usual care for depression. Participating practices were recruited from the AAFP NRN. Patient interview participants were recruited from the patients enrolled in the intervention arm via email invitation in the final weeks of their study involvement. Practice champion clinicians, study coordinators, and clinicians who cared for patients enrolled in the study were invited to participate postintervention.

### Ethical Considerations

This study was approved by the American Academy of Family Physicians Institutional Review Board (Protocol 20‐389). All participants consented to be in the study. However, those participants who took part in the intervention were sent an email asking if they would be willing to participate in an interview. Those who responded received an invitation for a scheduled interview. Prior to the start of the interview, a study team member thoroughly described participation: expected interview length, questions asked, permission to record, and participant rights (voluntary participation, can terminate participation at any time without penalty, remuneration, and possible risks). Risks, such as discomfort answering certain questions and the potential loss of anonymity and confidentiality, were explained. Verbal consent was then obtained. On completion of the interview, participants received compensation; a US $100 e-gift card was sent to their email. All identifying information was removed from interviews and study data to protect participant anonymity.

### Intervention

The Fora Health app supports goal setting and tracks daily medication adherence and side effects, weekly goal attainment, and behavioral health participation; assesses the 9-item Patient Health Questionnaire (PHQ-9) [22,23], 13-item Patient Activation Measure [24,25], World Health Organization-Five Well-Being Index [26,27], and Perceived Deficits Questionnaire [28,29] on a biweekly basis; and provides short educational tutorials about depression management on topics such as talking with your physician and goal setting. Patient participants began using the Primary Care Path app (powered by Fora Health) after meeting with the study coordinator to enroll and set goals. The Primary Care Path web interface allows staff and clinicians to see patient-reported data. The expectation was that the care team would use the information before visits or review between visits.

### Analysis

Separate semistructured interview guides were developed by the AAFP NRN study team for patient and care team participants, according to the RE-AIM (reach, effectiveness, adoption, implementation, and maintenance) evaluation framework [30-32]. The interview guides were reviewed and revised by the broader study team. Interviews were conducted from May to October 2021 by a family physician (AN) and 2 research project managers (AML and EAR), each with a master’s degree and training and experience in qualitative methods. Interviews were conducted online and lasted approximately 30 minutes, and were audio recorded and transcribed. Four research team members (MKF, AN, AML, and EAR) met frequently over 13 weeks to develop the codebook, using a priori and emergent codes. Meaning, some codes were generated based on the discussion guide, and others were developed through open coding, where codes emerge from the data. After the research team members agreed on the codes and their definitions, each transcript was coded once (MKF and EAR) using Atlas.ti.9 (Berlin and Germany), and an audit was conducted (AML) to ensure intercoder reliability. This inductive approach to data analysis was completed by 4 members of the research team (MKF, AN, AML, and EAR), using a combination of grounded theory and constant comparative methods [33,34]. Themes emerged through repeated reading of the data. Themes were discussed and agreed upon by the broader research team.

## Results

### Comparing Patient and Care Team Perspectives

The study team interviewed intervention patients (n=8) and intervention care team participants (lead clinicians, study coordinators, and other clinicians; n=8, shown in Tables1  2). The sample size was determined based on the 2 participant categories of the sampling frame and the sample’s sufficiency to provide a meaningful perspective and nuanced detail [35]. Data comparisons between the patient and care team participant strata showed similarities and differences for topics of app/interface usability (navigation and content and usability), tracking (medication adherence, side effects, goals, suggestions for additional tracking features, and communication), and program recommendations. Saturation was reached within the patient stratum as there was consistency across all patient data. Even though much of the data indicates overall agreement, additional explanations are provided where discordance is present. Example quotations are displayed at the end of each section.

**Table 1.** Patient demographics.

Patient participant	Sex	Race	Ethnicity	Year of birth	Employment status	Living situation	Treatments used for depression
1	Female	White	Non-Hispanic or Latinx	1997	Part time	With spouse or other family	Antidepressant medication and behavioral health/talk therapy
2	Female	White	Non-Hispanic or Latinx	1986	Full time	Alone	Antidepressant medication, behavioral health/talk therapy, and self-help strategies (books, apps, or other tools)
3	Female	White	Non-Hispanic or Latinx	1982	Part time	With spouse or other family	Antidepressant medication
4	Female	White	Non-Hispanic or Latinx	1988	Part time	With friends and/or roommates	Antidepressant medication
5	Female	White	Non-Hispanic or Latinx	1957	Part time	Alone	Self-help strategies (books, apps, or other tools)
6	Male	No response	No response	1964	Retired	With spouse or other family	Antidepressant medication and self-help strategies (books, apps, or other tools)
7	Male	White	Hispanic or Latinx	1969	Full time	With spouse or other family	Antidepressant medication
8	Male	White	Non-Hispanic or Latinx	1962	Part time	Alone	No response

**Table 2.** Care team demographics.

Care team participant	Sex	Race	Ethnicity	Birth year	Years in practice	Role/credential
1	Female	White	Non-Hispanic or Latinx	1993	1‐5	BH^a^ care team
2	Female	White	Non-Hispanic or Latinx	1970	16‐20	MD^b^
3	Female	White	Non-Hispanic or Latinx	1984	1‐5	BH care team, PhD^c^
4	Female	White	Non-Hispanic or Latinx	1991	1‐5	DO^d^
5	Male	White	Non-Hispanic or Latinx	1984	6‐10	MD
6	Male	Missing	Missing	Missing	Missing	Coordinator, Practice Manager
7	Male	White	Non-Hispanic or Latinx	1963	≥21	MD
8	Male	White	Non-Hispanic or Latinx	1989	6‐10	APRN^e^

a BH: behavioral health.

b MD: doctor of medicine.

c PhD: doctor of philosophy.

d DO: doctor of osteopathic medicine.

e APRN: advanced practice registered nurse.

### App/Interface Usability

#### Navigation

Overall, both patient and care team participants reported that the app/interface was simple and easy to use. Patient participants said entering data did not consume much time, making it practical to use in daily and weekly routines. They also liked the convenience of being able to enter data at any time. Care team participants stated that reporting, monitoring, viewing, and inputting information (eg, joint goals) in the interface was straightforward. Particularly, care team participants liked the red flag next to the patient ID for the suicidality alert for patients who responded positively to the PHQ-9 question 9, making it easy to identify which patients needed follow-up.

*I think how it emailed us about the [suicidality] alerts helped. Of course, as you would log on to the app, you would see that there was an alert on a specific patient’s chart and then being able to dismiss it once that concern was addressed.*

[Care team participant]

*Oh, it was definitely easy. It was very well designed, very clear. It never crashed. I never had an issue with it on my phone.*

[Patient participant]

#### Content and Usability

In terms of app content, patient participants thought the app was a dependable source of information. However, patient participants overwhelmingly stated they desired additional content such as videos and guides. They said the app was static, repetitive, and compliance-driven, with too much focus on medication adherence. They reported a desire for personalized insights regarding their own depression and targeted information on interpreting their results over time, as well as content options that evolve as their treatment changes. They also suggested displaying how much time an action takes to complete (watching videos, reading guides, etc). Care team participants who spoke with their patients specifically about the app responded similarly to patient participant impressions—that content was appropriate, but they wanted more substance. Care team participants also suggested expanding resources for patients beyond depression care and management to include topics such as substance use disorders and additional information for care team members, such as education on prescribing medications and using goal-setting techniques with patients. Some care team members liked that the program tenets of shared decision-making and goal setting were incorporated in the app/interface.

Some care team participants stated that the data and insights were helpful as they could monitor patients between visits via the interface. They said that seeing patterns and trends helped or could help facilitate conversations, particularly if a change was needed, such as medication, therapy, or goals. The residency practice specifically used the data in daily huddles to better prepare for appointments. At this practice, a few point people regularly logged into the interface, but many care team participants only reviewed printed reports. For those who did use the interface, a feature they appreciated was the ability to view the level of patient involvement and their activities, allowing insight into how patient participants were using and applying the tenets of the program. Some care team participants stated that they believed the content helped patients pay attention and be more mindful of their depression care and management; yet, they were also unsure if the data they viewed really contributed to better patient outcomes, treatment, or symptom management.

Care team participants appreciated seeing the results of the PHQ-9, which initiated a specific alert to a designated care team member if the patient responded positively to question 9 (suicidal ideation), particularly if this was not a concern at the previous visit. However, they thought patient outreach could be more efficient. For example, PHQ-9 information was not in the medical chart, so they had to create a note and send it to other care team members. Even though care team participants thought the PHQ-9 alerts were appropriate, they were concerned about patient truthfulness, thinking patient participants may not want to be contacted about their answers in the future, which could impact how they answered questions.

*My only thing that I couldn't figure out how to fix was every once in a while, there would be the small four-minute guides about setting goals with your caregiver, those ones. The last month and a half I was using it, it prompted me with the same guide every two days for a month and a half.*

[Patient participant]

*When it comes to features, about the only thing that I think would be useful is if there were any relevant articles, studies, videos or anything like that, that could be useful, that could be recommended or something like that. Just different ways to engage the user and to put more resources, basically.*

[Patient participant]

### Tracking

#### Medication Adherence

Patient participants who had never taken medication before stated that the daily medication question was particularly helpful, reporting that the daily check-in helped them think about goals and well-being more frequently. They reported being more mindful of their depression treatment and care throughout the week. However, for patient participants who already had a routine for taking medication, the reminders were not particularly helpful. Care team participants reiterated that for patients who need help establishing routines, reminders seemed to help with medication adherence.

*I think it’s really helpful if you’re not good at taking your medication. I think that that is the number—I take it every night before bed, so I’m pretty strict in that routine. I can see that if it’s something you’re not used to doing, it being really helpful for that. It was really simple to use. If there were older users that weren’t comfortable with technology, it would be really easy to navigate for them.*

[Patient participant]

Overall, patient participants liked the option for tracking medication use, as it showed day-to-day adherence. Some patient participants viewed even small changes in their depression symptoms as motivation to continue taking medication. However, some patient participants did not track their medication use, or they only tracked it for a short period of time because they either already took medications consistently, lacked motivation to track, or were not interested in tracking. Patient and care team participants thought the act of tracking medication use contributed to a sense of accountability in depression treatment and care. However, patient participants mentioned that when they switched medications or changed dosage, it was difficult to account for those changes in the app. They also wanted questions streamlined to save time and avoid repetition. Finally, some care team participants questioned whether this approach changed medication adherence.

*I think the biggest way it [tracking] probably affected their treatment was just actually (a) holding them a bit more accountable for what they were trying to do on their end, but (b) just seeing them check in daily with themselves or however often they were doing it*...*I think being able to be a little more retrospective more frequently helped them gain more insight too into how they were doing.*

[Care team participant]

*No, it [the program] didn’t look like it [changed medication adherence]. It looked like we saw the same things. Some people were consistently taking their meds, some people who had some side effects stopped without telling us and would show up for the next visit. That didn’t look like it changed much.*

[Care team participant]

#### Side Effects

Tracking side effects was reported to be particularly helpful as patient participants often were not confident in differentiating between medication side effects and depression-related symptoms. For some patient participants, tracking side effects led to new discussions with their care team or therapist. Some care team participants stated they responded or could respond more quickly to side effects because patients were tracking them in the app, stating this was the most useful data collected because of in-depth, near real-time information, which allowed for timely adjustment of medications. Furthermore, in the residency practice, the data were used to help resident physicians make care decisions.

*They really like the fact that if they were experiencing side effects that they could note it in the app, and their care team would be able to see it sooner before their next visit. It also allowed us to be aware of what’s going on and make necessary changes if we needed to, if the side effects were that uncomfortable, or just to see if their PHQ was increasing that a change needed to be made.*

[Care team participant]

*It’d make you cognizant of the fact that it’s something that you need to monitor, need to stay on top of. I think what it really helps is when it comes—’cause it makes you think about any side effects you my have had, and you may be having side effects but not necessarily attribute ’em to what you’re takin’ medication for, so it’s a way of makes you think, and it’s like, you know what? I did have this today, or I did go do that. Did that have something to do with it? At very least, it makes you think about.*

[Patient participant]

#### Goals

In general, both patient and care team participants found value in setting and tracking goals. Patient participants stated it was helpful to set simple and reachable goals, and they could apply goal setting to other areas in their depression self-management beyond medication adherence (eg, exercising, spending time with family, reading, etc). In addition, patient participants appreciated seeing their progress displayed concretely in the app. Care team participants also thought that the tracking helped facilitate patient participants’ depression self-management. Some care team participants reported that they had used goal setting as part of their normal care prior to study involvement, but the app/interface further facilitated the process of setting and tracking goals. They perceived the program, via the app/interface, as providing structure and consistency to ensure that goal setting was incorporated into discussions with patients. One care team participant stated that when patient participants used the app, it made conversations about setting goals easier, and it helped patient and care team participants to be on the same page.

Patient and care team participants also noted some hurdles with the goal-setting features. Patient participants discussed the difficulty of tracking goals within the app, including confusing responses (eg, goal benchmarks that did not make sense due to care team user error) or insufficient goal attainment responses. They wanted the ability to modify goals or remove outdated goals. Some patient participants reported feeling worse when reminded of a goal they were not meeting or that was no longer aligned with their current plan. Care team participants agreed that patient participants had difficulty managing goals within the app regarding frequency, milestones, and attainment selection.

*Like I said, my favorite part of it is that you guys have those goals on there, which is typically something that I really like to do with patients and engaging them in shared decision-making, problem-solving with them, and goal setting to help accomplish their goals.*

[Care team participant]

*Yeah, one of our patient’s weight was a big thing tied to her depression. She felt like the more she gained weight, the more depressed she felt. One of the things that really helped her was making goals in terms of exercising. She said having the goal of exercise by walking outside three times a week definitely really helped her become more engaged. She did lose, I believe, about 10 pounds, 15 pounds, something around there, which also improved her mood as well ’cause she was seeing that she was making these different changes.*

[Care team participant]

*That [questions about goals] was a little bit difficult to answer because my goal was to read twice a week for 15 minutes*. *The language of the app was, “Did you complete it once or did you complete it twice?” I wasn’t sure because I was doing it twice already, should I say I did it once or should I say I did it twice? Sometimes just due to the language of the app, it didn’t fit exactly what I was trying to do. Maybe a better way to see your progress with goals, rather than just checking off that you did it, maybe something like that.*

[Patient participant]

#### Suggestions for Additional Tracking Features

Patient participants suggested streamlining questions to save time and avoid repetition. They also wanted to add some additional tracking features (eg, other self-management behavior that did not involve medication, such as physical activity, emotions, or well-being, with a place to write notes). Some desired a way to record more details on their progress. Care team participants said it was difficult to interpret the accuracy of the log (eg, medication compliance) and incomplete information (eg, missing data could indicate noncompliance or forgetfulness).

#### Communication

Patient and care team participants expressed that tracking encouraged or had the potential to encourage communication with one another. Patient participants liked the possibility of checking in with care team participants, especially without an appointment. Some felt that inputting data prior to appointments made the time together more efficient. Some care team participants also thought the appointments were easier and more effective when patients had tracked medication and goals. Care team participants stated that tracking enabled them to reference data before or during visits, highlight potential concerns, reassure alignment with the patient, and use information in follow-up calls. Some patient participants thought that the app had no impact on shared decision-making for depression treatment and management. Other patient participants stated it had made a difference, although some were unsure if a care team member viewed their information beforehand. Some patient and care team participants thought tracking impacted their relationship with one another; tracking reinforced feelings that the care team was invested in patient well-being, and the patient was invested in their own care. Care team participants thought that patient participants were more involved and proactive in their own care, and some believed patient engagement improved in the process.

*I don’t know that it cured the symptoms or anything like that. I just think that it was helpful and that I can keep that communication going with my team, so they knew what was going on.*

[Patient participant]

*This provided data points in between visits, especially while we’re titrating medications because often we’re starting a new [medication], changing something, changing a dosing, and this allowed communication points in between those visits or ways to track that data outside of these. We often will have them come back in six weeks after a medication change has been made. This can allow for interim information to help making a decision moving forward too.*

[Care team participant]

### App Use Recommendations

Patient and care team participants believed using the app could benefit several groups, namely those who are new to taking or switching medications, have trouble remembering to take medications, are proactive in their care, want to make a behavioral change, need reminders, need accountability, and are seeking increased adherence. Some care team participants recommended targeting patients who needed closer follow-up and thought the app was more beneficial for those who were more diligent about their depression care and management, as the increased engagement gave them better insight into their health. Some care team participants indicated that using the app interface did not impact or change setting goals, reaching goals, making shared decisions, or patient outcomes. Reasons provided were either that their practice had implemented a patient-centered approach previously or that they needed data to compare before and after study participation to make a true assessment.

Care team participants thought the workflow around the interface could be improved. They overwhelmingly stated the need for electronic health record (EHR) integration because logging into the interface interrupted workflow, making it impractical during a patient appointment. Some care team participants admitted they did not access patient participant information: logging in was not well integrated into their workflow, they did not remember to log in, or they did not know how to log in. For example, one care team participant in the residency practice commented that it would have been helpful for the attending physician to see the data, and one patient participant stated that they wanted to share data with a psychiatrist outside of their primary care practice. Furthermore, some care team participants spoke about the need for app support within the practice. One care team participant stated that the practice had a dedicated behavioral health care specialist who carried out nonmedication interventions, and much of the program fell under their purview (eg, providing information to physicians and following up with patients). It was stressed that behavioral health is a team effort, and office staff are needed for integration.

Overall, patient participants who tracked and care team participants who used data to inform conversations saw value in shared decision-making and setting goals and thought these tenets could be applied to other conditions, such as anxiety, weight management, diabetes, high blood pressure, attention deficit disorder or attention-deficit/hyperactivity disorder, and medication compliance.

*It really is about the EMR [electronic medical record], just because I’m spending so much time on it every day. It’s always gonna be hard for me to have to login into something else outside of that when everything else is integrated.*

[Care team participant]

## Discussion

### Principal Findings

Through this study, we identified barriers to and facilitators for implementation of a pilot program that incorporates a patient-facing mobile app and care team web interface to support depression treatment and management in primary care practices. These results on patient and care team participant perspectives of program implementation contribute to the rapidly expanding field of the use of digital health for depression care [17,36-45]. While some results between patient and care team participant strata were rather uniform, others differed. Our findings indicate that tracking medication adherence, side effects, and goals could allow both patient and care team participants greater insight into depression treatment and management. However, though the app may have helped some patient participants compile information for tracking, which may have led to more focused visits, not all care team participants thought app use improved patient outcomes. This may be more a matter of perception—perhaps patient participants who engaged regularly with the app thought they had better outcomes, possibly related to the perception of having more connectivity with their care team. Care team participants reported they could not definitively determine whether the app and program impacted patient outcomes, patient relationships, shared decision-making, goal setting, or goal attainment without tangible pre- and postintervention data. Nonetheless, medication adherence and goal setting are often related to depression outcomes and can be challenging [46-48]; this program could be another tool in their toolbox. Practices that already have patient-centered approaches to care may find aspects of this program duplicative; yet practices that do not prioritize approaches to goal setting and shared decision-making may experience more benefits if implementing this program, or a similar one.

### Data and Action

App data in and of itself may not impact patient outcomes; it may be that the increased follow-up and touchpoints promote improvement in depression symptoms, as is evident in the literature [49,50]. The program was designed to connect patient participant data to the care team, allowing the care team to get real-time insight into patient symptoms and functioning between appointments. Thus, care team members who receive and review app data may be better able to recommend follow-up, suggest care changes, inform conversations, and shape next steps versus those who do not use additional data points collected outside of a care visit. The novel contribution of this study is that care team members who viewed near real-time information had an opportunity to make more timely decisions, especially for medication changes. Therefore, data insights provided to the care team via the interface may allow treatment pivots based on more accurate data on what is or is not working. This finding suggests that those care team members who accessed patient information in near real-time could make more prompt evidence-based decisions, which is particularly important for a patient with depression symptoms that impair day-to-day functioning.

### Program Implementation

Even though care team participants appreciated aspects of the program, they agreed that successful implementation would need to be more integrated into existing workflows. It is unclear to what extent care team participants, especially physicians, accessed data prior to and during visits, and they often said that accessing a separate website was too time-consuming during a busy clinical day. This is consistent with other literature that demonstrates that app data integration into clinical workflows is often a requirement for use due to heavy workloads [51-55]. EHR integration would allow all care team members access, a seamless transition for viewing data, and a comprehensive view of patient data. For example, if a patient scheduled an appointment for something unrelated to depression, a care team member would not necessarily review their depression data on a separate website. It is well documented that EHRs can help clinicians prepare for appointments, making them more efficient [56,57].

For uptake of this program, other workflow processes may need to be established depending on the practice, such as the level of behavioral health integration or resource support (eg, access to behavioral health professionals or referrals to community resources) [58-61]. Another consideration is designing the best approach for using data in conversations with patients [17]. Future iterations of this program could include additional education for care team members regarding roles, recommendations, and patient interactions.

### Limitations

This study has limitations. First, our study sample was small and was racially and ethnically homogeneous, limiting the generalizability of the findings. Second, data saturation was reached in the patient stratum but not the care team member stratum. The care team contained diverse roles. We interviewed 8 primary care physicians and clinicians; their experiences varied. For example, a behavioral health professional or care coordinator may have had a touchpoint with the patient, and physicians may or may not have used the interface. Therefore, it is inconclusive as to whether physicians accessed and used the data before or during visits. To help remedy this situation, care team participants recommended embedding the interface data into the EHR. Third, to some extent, these data were influenced by patient and care team member engagement with the program. For example, if a patient used the app for a limited time span, they may not have tracked their behaviors long enough to see trends or for care team participants to make recommendations. Finally, a patient’s depression cycle may have affected app use and program involvement. If depression symptoms were more prominent, patient participants may not have had the motivation to track medication adherence, side effects, or goals.

### Conclusions

Implementation of a digital health program for depression treatment and management in primary care practices may help support patient medication adherence, facilitate timely medication changes, encourage goal setting, and foster communication between patients and care team members. While patient and care team participants valued program tenets, enhancements such as minimizing workflow interruptions, integrating data into the EHR, providing education and best practices for patient interactions, augmenting content with helpful resources, and adding personalized content that evolves alongside patient progression could increase program engagement.

## Abbreviations

AAFP NRN: American Academy of Family Physicians National Research Network
EHR: electronic health record
PHQ-9: 9-item Patient Health Questionnaire
RE-AIM: reach, effectiveness, adoption, implementation, and maintenance

## References

[1] Ettman CK, Abdalla SM, Cohen GH, Sampson L, Vivier PM, Galea S (2020). Prevalence of depression symptoms in US adults before and during the COVID-19 pandemic. JAMA Netw Open.

[2] Hunter CL, Goodie JL, Oordt MS, Dobmeyer AC (2009). Integrated Behavioral Health in Primary Care: Step-by-Step Guidance for Assessment and Intervention.

[3] Jackson JL, Kuriyama A, Bernstein J, Demchuk C (2022). Depression in primary care, 2010-2018. Am J Med.

[4] Chang ET, Magnabosco JL, Chaney E (2014). Predictors of primary care management of depression in the Veterans Affairs healthcare system. J Gen Intern Med.

[5] Chew-Graham CA, Mullin S, May CR, Hedley S, Cole H (2002). Managing depression in primary care: another example of the inverse care law?. Fam Pract.

[6] Egede LE (2007). Failure to recognize depression in primary care: issues and challenges. J Gen Intern Med.

[7] Blackstone SR, Sebring AN, Allen C, Tan JS, Compton R (2022). Improving depression screening in primary care: a quality improvement initiative. J Community Health.

[8] Garcia ME, Hinton L, Neuhaus J, Feldman M, Livaudais-Toman J, Karliner LS (2022). Equitability of depression screening after implementation of general adult screening in primary care. JAMA Netw Open.

[9] Kyanko KA, A Curry L, E Keene D, Sutherland R, Naik K, Busch SH (2022). Does primary care fill the gap in access to specialty mental health care? a mixed methods study. J Gen Intern Med.

[10] Samples H, Stuart EA, Saloner B, Barry CL, Mojtabai R (2020). The role of screening in depression diagnosis and treatment in a representative sample of US primary care visits. J Gen Intern Med.

[11] Dineen-Griffin S, Garcia-Cardenas V, Williams K, Benrimoj SI (2019). Helping patients help themselves: a systematic review of self-management support strategies in primary health care practice. PLoS ONE.

[12] Kappelin C, Carlsson AC, Wachtler C (2022). Specific content for collaborative care: a systematic review of collaborative care interventions for patients with multimorbidity involving depression and/or anxiety in primary care. Fam Pract.

[13] Menear M, Duhoux A, Roberge P, Fournier L (2014). Primary care practice characteristics associated with the quality of care received by patients with depression and comorbid chronic conditions. Gen Hosp Psychiatry.

[14] Pung A, Fletcher SL, Gunn JM (2018). Mobile app use by primary care patients to manage their depressive symptoms: qualitative study. J Med Internet Res.

[15] Lattie EG, Adkins EC, Winquist N, Stiles-Shields C, Wafford QE, Graham AK (2019). Digital mental health interventions for depression, anxiety, and enhancement of psychological well-being among college students: systematic review. J Med Internet Res.

[16] Wang K, Varma DS, Prosperi M (2018). A systematic review of the effectiveness of mobile apps for monitoring and management of mental health symptoms or disorders. J Psychiatr Res.

[17] McCue M, Blair C, Fehnert B (2022). Mobile app to enhance patient activation and patient-provider communication in major depressive disorder management: collaborative, randomized controlled pilot study. JMIR Form Res.

[18] Rathbone AL, Prescott J (2017). The use of mobile apps and SMS messaging as physical and mental health interventions: systematic review. J Med Internet Res.

[19] Milne-Ives M, Lam C, De Cock C, Van Velthoven MH, Meinert E (2020). Mobile apps for health behavior change in physical activity, diet, drug and alcohol use, and mental health: systematic review. JMIR Mhealth Uhealth.

[20] Carlo AD, Hosseini Ghomi R, Renn BN, Areán PA (2019). By the numbers: ratings and utilization of behavioral health mobile applications. NPJ Digit Med.

[21] Torous J, Andersson G, Bertagnoli A (2019). Towards a consensus around standards for smartphone apps and digital mental health. World Psychiatry.

[22] Kroenke K, Spitzer RL, Williams JB (2001). The PHQ-9: validity of a brief depression severity measure. J Gen Intern Med.

[23] Kroenke K, Spitzer RL (2002). The PHQ-9: a new depression diagnostic and severity measure. Psychiatr Ann.

[24] Hibbard JH, Mahoney ER, Stockard J, Tusler M (2005). Development and testing of a short form of the patient activation measure. Health Serv Res.

[25] (2021). Patient Activation Measure® (PAM®): learn more about the leading assessment of patient activation. Insignia Health.

[26] Topp CW, Østergaard SD, Søndergaard S, Bech P (2015). The WHO-5 Well-Being Index: a systematic review of the literature. Psychother Psychosom.

[27] (1998). Wellbeing measures in primary health care/the depcare project. https://iris.who.int/server/api/core/bitstreams/8af98105-30ec-4da4-9fe9-097f7459e6da/content.

[28] Sullivan MJ, Edgley K, Dehoux E (1990). A survey of multiple sclerosis: I. Perceived cognitive problems and compensatory strategy use. Can J Rehabil.

[29] Lam RW, Lamy FX, Danchenko N (2018). Psychometric validation of the Perceived Deficits Questionnaire-Depression (PDQ-D) instrument in US and UK respondents with major depressive disorder. Neuropsychiatr Dis Treat.

[30] Klesges LM, Estabrooks PA, Dzewaltowski DA, Bull SS, Glasgow RE (2005). Beginning with the application in mind: designing and planning health behavior change interventions to enhance dissemination. Ann Behav Med.

[31] Glasgow RE, Vogt TM, Boles SM (1999). Evaluating the public health impact of health promotion interventions: the RE-AIM framework. Am J Public Health.

[32] Gaglio B, Shoup JA, Glasgow RE (2013). The RE-AIM framework: a systematic review of use over time. Am J Public Health.

[33] Singer M, Baer H (2012). Introducing Medical Anthropology: A Discipline in Action.

[34] Patton MQ (1990). Qualitative Evaluation and Research Methods.

[35] Corbin JM, Strauss AL (2014). Basics of Qualitative Research: Techniques and Procedures for Developing Grounded Theory.

[36] Serrano-Ripoll MJ, Zamanillo-Campos R, Fiol-DeRoque MA, Castro A, Ricci-Cabello I (2022). Impact of smartphone app-based psychological interventions for reducing depressive symptoms in people with depression: systematic literature review and meta-analysis of randomized controlled trials. JMIR Mhealth Uhealth.

[37] Graham AK, Greene CJ, Kwasny MJ (2020). Coached mobile app platform for the treatment of depression and anxiety among primary care patients: a randomized clinical trial. JAMA Psychiatry.

[38] Leong QY, Sridhar S, Blasiak A (2022). Characteristics of mobile health platforms for depression and anxiety: content analysis through a systematic review of the literature and systematic search of two app stores. J Med Internet Res.

[39] McCloud T, Jones R, Lewis G, Bell V, Tsakanikos E (2020). Effectiveness of a mobile app intervention for anxiety and depression symptoms in university students: randomized controlled trial. JMIR Mhealth Uhealth.

[40] Deady M, Glozier N, Calvo R (2022). Preventing depression using a smartphone app: a randomized controlled trial. Psychol Med.

[41] Broglia E, Millings A, Barkham M (2019). Counseling with guided use of a mobile well-being app for students experiencing anxiety or depression: clinical outcomes of a feasibility trial embedded in a student counseling service. JMIR Mhealth Uhealth.

[42] Teepe GW, Da Fonseca A, Kleim B (2021). Just-in-time adaptive mechanisms of popular mobile apps for individuals with depression: systematic app search and literature review. J Med Internet Res.

[43] Myers A, Chesebrough L, Hu R, Turchioe MR, Pathak J, Creber RM (2020). Evaluating commercially available mobile apps for depression self-management. AMIA Annu Symp Proc.

[44] Lu SC, Xu M, Wang M (2022). Effectiveness and minimum effective dose of app-based mobile health interventions for anxiety and depression symptom reduction: systematic review and meta-analysis. JMIR Ment Health.

[45] Zhang R, Nicholas J, Knapp AA (2019). Clinically meaningful use of mental health apps and its effects on depression: mixed methods study. J Med Internet Res.

[46] Bosworth HB, Voils CI, Potter GG, Steffens DC (2008). The effects of antidepressant medication adherence as well as psychosocial and clinical factors on depression outcome among older adults. Int J Geriatr Psychiatry.

[47] Coote HMJ, MacLeod AK (2012). A self-help, positive goal-focused intervention to increase well-being in people with depression. Clin Psychol Psychother.

[48] Jacob J, Stankovic M, Spuerck I, Shokraneh F (2022). Goal setting with young people for anxiety and depression: what works for whom in therapeutic relationships? A literature review and insight analysis. BMC Psychol.

[49] Simon GE, Ralston JD, Savarino J, Pabiniak C, Wentzel C, Operskalski BH (2011). Randomized trial of depression follow-up care by online messaging. J Gen Intern Med.

[50] Solberg LI, Trangle MA, Wineman AP (2005). Follow-up and follow-through of depressed patients in primary care: the critical missing components of quality care. J Am Board Fam Pract.

[51] Huckvale K, Nicholas J, Torous J, Larsen ME (2020). Smartphone apps for the treatment of mental health conditions: status and considerations. Curr Opin Psychol.

[52] Dinkel D, Harsh Caspari J, Fok L (2021). A qualitative exploration of the feasibility of incorporating depression apps into integrated primary care clinics. Transl Behav Med.

[53] Cohen DJ, Keller SR, Hayes GR, Dorr DA, Ash JS, Sittig DF (2016). Integrating patient-generated health data into clinical care settings or clinical decision-making: lessons learned from project HealthDesign. JMIR Hum Factors.

[54] Ye J (2021). The impact of electronic health record-integrated patient-generated health data on clinician burnout. J Am Med Inform Assoc.

[55] Gordon WJ, Landman A, Zhang H, Bates DW (2020). Beyond validation: getting health apps into clinical practice. NPJ Digit Med.

[56] O’Malley AS, Draper K, Gourevitch R, Cross DA, Scholle SH (2015). Electronic health records and support for primary care teamwork. J Am Med Inform Assoc.

[57] Sinsky CA, Sinsky TA, Rajcevich E (2015). Putting pre-visit planning into practice. Fam Pract Manag.

[58] Moon K, Sobolev M, Kane JM (2022). Digital and mobile health technology in collaborative behavioral health care: scoping review. JMIR Ment Health.

[59] Moon KC, Sobolev M, Grella M, Alvarado G, Sapra M, Ball T (2022). An mHealth platform for augmenting behavioral health in primary care: longitudinal feasibility study. JMIR Form Res.

[60] Hoffman L, Benedetto E, Huang H (2019). Augmenting mental health in primary care: a 1-year study of deploying smartphone apps in a multi-site primary care/behavioral health integration program. Front Psychiatry.

[61] Carleton KE, Patel UB, Stein D, Mou D, Mallow A, Blackmore MA (2020). Enhancing the scalability of the collaborative care model for depression using mobile technology. Transl Behav Med.

